# Genetic screening reveals cone cell-specific factors as common genetic targets modulating rival-induced prolonged mating in male *Drosophila melanogaster*

**DOI:** 10.1093/g3journal/jkae255

**Published:** 2024-11-04

**Authors:** Yanying Sun, Xiaoli Zhang, Zekun Wu, Wenjing Li, Woo Jae Kim

**Affiliations:** The HIT Center for Life Sciences, Harbin Institute of Technology, Harbin, Heilongjiang 150006, China; The HIT Center for Life Sciences, Harbin Institute of Technology, Harbin, Heilongjiang 150006, China; The HIT Center for Life Sciences, Harbin Institute of Technology, Harbin, Heilongjiang 150006, China; The HIT Center for Life Sciences, Harbin Institute of Technology, Harbin, Heilongjiang 150006, China; The HIT Center for Life Sciences, Harbin Institute of Technology, Harbin, Heilongjiang 150006, China; Medical and Health Research Institute, Zhengzhou Research Institute of HIT, Zhengzhou, Henan 450000, China

**Keywords:** cone cells, mating duration, interval timing behavior, *Drosophila*, aggressive behavior, visual pathway, *CG10026/macewindu*, non-neuronal regulation, *CrzR*, *Cyp6a20*, *Cyp4d21*

## Abstract

Male–male social interactions exert a substantial impact on the transcriptional regulation of genes associated with aggression and mating behavior in male *Drosophila melanogaster*. Throughout our comprehensive genetic screening of aggression-related genes, we identified that the majority of mutants for these genes are associated with rival-induced and visually oriented mating behavior, longer-mating duration (LMD). The majority of mutants with upregulated genes in single-housed males significantly altered LMD behavior but not copulation latency, suggesting a primary regulation of mating duration. Single-cell RNA-sequencing revealed that LMD-related genes are predominantly co-expressed with male-specific genes like *dsx* and *Cyp6a20* in specific cell populations, especially in cone cells. Functional validation confirmed the roles of these genes in mediating LMD. Expression of LMD genes like *Cyp6a20*, *Cyp4d21*, and *CrzR* was enriched in cone cells, with disruptions in cone cell-specific expression of *CrzR* and *Cyp4d21* leading to disrupted LMD. We also identified a novel gene, *CG10026/Macewindu*, that reversed LMD when overexpressed in cone cells. These findings underscore the critical role of cone cells as a pivotal site for the expression of genes involved in the regulation of LMD behavior. This study provides valuable insights into the intricate mechanisms underlying complex sexual behaviors in *Drosophila.*

## Introduction

Social interactions play a pivotal role in shaping both aggression and mating behaviors in *Drosophila melanogaster* (Sokolowski 2010; Billeter and Levine 2013; Schneider *et al*. 2017; Chen and Sokolowski 2022). These interactions can significantly impact the expression of aggressive behavior (Yuan *et al*. 2014), as well as influence mating duration (MD) and reproductive success (Kim *et al*. 2012, 2013; Lee *et al*. 2023). In the context of aggression, social experiences have been shown to suppress aggressive behavior in *Drosophila*. For instance, flies that are group-housed exhibit reduced aggression compared to those that are isolated. This suggests that social interactions can modulate aggressive behavior by altering the sensitivity to pheromones or by modifying the activity of specific genes associated with aggression (Ueda and Kidokoro 2002; Wang and Anderson 2010; Ramin *et al*. 2014; Yuan *et al*. 2014; Kravitz and Fernandez 2015). One such gene is *Cyp6a20*, which encodes a cytochrome P450 enzyme (Wang *et al*. 2008). Increased expression of *Cyp6a20* under the influence of social experience has been linked to a decrease in aggression. Moreover, mutations in *Cyp6a20* lead to enhanced aggression in group-housed flies, indicating that social interactions can act through this gene to regulate aggressive behavior.

Similarly, social interactions also play a crucial role in shaping mating behavior in *Drosophila* (Krupp *et al*. 2008; Billeter and Levine 2013; Chen and Sokolowski 2022). In the context of mating behavior, the duration of copulation or mating duration in *Drosophila* is a complex trait influenced by a variety of genetic and environmental factors. Mating duration is crucial for reproductive success and can be modified by social interactions, such as the presence of rival males (Bretman *et al*. 2009; Kim *et al*. 2012). Group-housed males have been observed to exhibit longer-mating durations (LMD) compared to isolated males. This prolonged mating duration in group-housed males may be attributed to increased mating competitiveness or changes in the expression of genes associated with mating behavior (Bretman, Gage, *et al*. 2011). Social interactions can influence the activity of these genes, thereby modulating mating duration and reproductive success.

The study of aggression-related genes in the context of mating duration may provide insights into the genetic targets that mediate the effects of social competition on reproductive strategies (Tinbergen 1968; Holekamp and Strauss 2016). To investigate this further, we selected several genetic factors based on the microarray data from a previous study that identified targets associated with aggressive behavior under conditions similar to those that lead to LMD in *Drosophila* (Wang *et al*. 2008). Wang *et al*. conducted a microarray analysis to discern genes that are differentially expressed between singly reared males and males reared in groups with conspecific rivals for a period of 3–7 days (Wang *et al*. 2008). This experimental condition corresponds precisely to the scenario in which males exhibit distinct mating duration behaviors, which we have designated as LMD behavior (Bretman *et al*. 2009; Kim *et al*. 2012). Drawing on this correspondence, we hypothesize that the genes identified as differentially regulated in the study by Wang *et al*. may also influence LMD behavior.

Here, we report the results of our genetic screening to identify common genetic targets affecting rival-induced LMD behavior. Our findings reveal cone cell-specific factors as key players in the modulation of mating duration in response to social competition. In the compound eye of *Drosophila*, the cone cell plays a role analogous to that of glial cells supporting photoreceptors. Much like how glia provide nourishment, maintain homeostasis, and offer structural support to neurons in the nervous system, cone cells in the *Drosophila* eye act in a supportive capacity to the photoreceptor cells, which are responsible for capturing light and initiating the visual process (Vernadakis 1996). Cone cells are crucial for visually oriented behavior in Drosophila, as they are involved in the processing and integration of visual information. They help in the discrimination of colors and the perception of light intensity, which are essential for tasks such as navigation, mate selection, and foraging. Since LMD behavior is also highly dependent on visually oriented inputs, these results contribute to a deeper understanding of the complex interplay between genetics and social environment in shaping reproductive behaviors and may provide a foundation for future studies on the molecular mechanisms underlying the evolution of mating strategies in *Drosophila* (Fernández and Kravitz 2013).

## Materials and methods

### Fly rearing and strains

*Drosophila melanogaster* were raised on cornmeal-yeast medium at similar densities to yield adults with similar body sizes. Flies were kept in 12 h light: 12 h dark cycles (LD) at 25°C (ZT 0 is the beginning of the light phase, ZT12 beginning of the dark phase). All mutants and transgenic lines used here have been described previously. The following lines were obtained from Bloomington Stock Center (#stock number): *spin*^*EY08566*^ (#16431), *bgm*^*1*^ (#28120), *wdp*^*A391*^ (#16105), *Pisd^EY03559^* (#15442), *Jhe*^*1*^ (#19293), *Nplp1*^*EY1108*^ (#20253), *speck^lo^* (#3193), *RYa-R*^*MB09874*^ (#29070), *CAP*^*49e*^ (#265278), *pkg21D^MB04805^* (#24228), *Hexo1*^*e00001*^(#17805), *CG1544*^*MB04693*^ (#24216), *CG3036*^*EY00382*^ (#14839), *CG10026*^*d02517*^ (#19177), *CG12560*^*MI11224*^ (#29186), *CG15093*^*d04924*^ (#19211), *CG31689*^*DG12405*^ (#20596), *CG31075*^*G7020*^ (#30176), *CG33120*^*MB07596*^ (#25645), *CrzR*^*MB00838*^ (#22910), *Cyp6a20*^*e02611*^ (#85145), *Cyp4d21*^*MB11663*^ (#29238), *ebony*^*1*^ (#1658), *fbl*^*1*^ (#11777), *Pri^1^* (#560), *CG8654*^*MB09631*^ (#26154), *CG11200*^*d02302*^ (#19173), *UAS-CrzR-RNAi* (#26017), *elav*^*c155*^; *UAS-Dcr-2.D* (#25750), *Df^Exel6234^* (#7708), *repo-GAL4* (#7415)*, pros-GAL4*, (#80572), *b6*^*EPG352*^ (#26590), *GMR-GAL4* (#9146), *orco-GAL4* (*#23129)*, *Crys*^*MI07191*^(#43607), *UAS-CD4tdGFP*(#35839), and *UAS-RedStinger*(#8546).

The following lines were obtained from Tsinghua RNAi Stock (#stock number): *spin-RNAi* (#2788), *Cyp6a20-RNAi* (#4635.N), and *retinin-RNAi* (#3345.N)

The following lines were obtained from Kyoto Stock Center (#stock number): *spin*^*NP3138*^*-GAL4* (#104388).

### Mating duration assays for successful copulation

The mating duration assay in this study has been reported (Kim *et al*. 2012, 2013; Lee *et al*. 2023). To enhance the efficiency of the mating duration assay, we utilized the *Df(1)^Exel6234^* (DF here after) genetic modified fly line in this study, which harbors a deletion of a specific genomic region that includes the sex peptide receptor (SPR) (Parks *et al*. 2004; Yapici *et al*. 2008). Previous studies have demonstrated that virgin females of this line exhibit increased receptivity to males (Yapici *et al*. 2008). We conducted a comparative analysis between the virgin females of this line and the Canton-S virgin females and found that both groups induced shorter mating duration (SMD). Consequently, we have elected to employ virgin females from this modified line in all subsequent studies. For naïve males, 40 males from the same strain were placed into a vial with food for 5 days. For single reared males, males of the same strain were collected individually and placed into vials with food for 5 days. For experienced males, 40 males from the same strain were placed into a vial with food for 4 days and then 80 DF virgin females were introduced into vials for the last 1 day before assay. 40 DF virgin females were collected from bottles and placed into a vial for 5 days. These females provide both sexually experienced partners and mating partners for mating duration assays. On the fifth day after eclosion, males of the appropriate strain and DF virgin females were mildly anesthetized by CO_2_. After placing a single female into the mating chamber, we inserted a transparent film and then placed a single male on the other side of the film in each chamber. After allowing for 1 h of recovery in the mating chamber in 25°C incubators, we removed the transparent film and recorded the mating activities. Only those males that succeeded in mating within 1 h were included for analyses. Initiation and completion of copulation were recorded with an accuracy of 10 sec, and total mating duration was calculated for each couple. Genetic controls with *GAL4/+* or *UAS/+* lines were omitted from Supplementary figures, as prior data confirm their consistent exhibition of normal LMD and SMD behaviors (Kim *et al*. 2012, 2013; Lee *et al*. 2023; Huang *et al*. 2024; Zhang *et al*. 2024). Hence, genetic controls for LMD and SMD behaviors were incorporated exclusively when assessing novel fly strains that had not previously been examined. In essence, internal controls were predominantly employed in the experiments, as LMD and SMD behaviors exhibit enhanced statistical significance when internally controlled. Within the LMD assay, both group and single conditions function reciprocally as internal controls. A significant distinction between the naïve and single conditions implies that the experimental manipulation does not affect LMD. Conversely, the lack of a significant discrepancy suggests that the manipulation does influence LMD. In the context of SMD experiments, the naïve condition (equivalent to the group condition in the LMD assay) and sexually experienced males act as mutual internal controls for one another. A statistically significant divergence between naïve and experienced males indicates that the experimental procedure does not alter SMD. Conversely, the absence of a statistically significant difference suggests that the manipulation does impact SMD. Hence, we incorporated supplementary genetic control experiments solely if they were deemed indispensable for testing. All assays were performed from noon to 4 Pm . We conducted blinded studies for every test.

### Courtship assays for copulation latency (CL) and courtship index (CI)

Courtship assay was performed as previously described (Griffith and Ejima 2009b) under normal light conditions in circular courtship arenas 11 mm in diameter, from noon to 4 Pm . We utilized the time at which copulation (mating) was initiated to quantify copulation latency (CL). The statistical methodology applied for the analysis of CL is identical to that employed for the MD assay. Upon the onset of courtship behavior, the courtship index (CI) was computed as the proportion of time a male dedicated to courtship-related behaviors within a 10-minute observation window or until the initiation of mating. The initiation of mating is defined as the moment at which male flies successfully achieve mounting on females.

### Single-nucleus RNA-sequencing analyses

The snRNAseq dataset analyzed in this paper is published by Li *et al.* (2022) and available at the Nextflow pipelines (VSN, https://github.com/vib-singlecell-nf), the availability of raw and processed datasets for users to explore, and the development of a crowd-annotation platform with voting, comments, and references through SCope (https://flycellatlas.org/scope), linked to an online analysis platform in ASAP (https://asap.epfl.ch/fca). For the generation of the tSNE plots, we utilized the Fly SCope website (https://scope.aertslab.org/#/FlyCellAtlas/*/welcome). Within the session interface, we selected the appropriate tissues and configured the parameters as follows: “Log transform” enabled, “CPM normalize” enabled, “Expression-based plotting” enabled, “Show labels” enabled, “Dissociate viewers” enabled, and both “Point size” and “Point alpha level” set to maximum. For the “Head” and “Body” tissues, we accessed the respective “Head” or “Body” sessions within the “10X” RNAseq dataset. For all other tissues, we referred to the individual tissue sessions within the “10X Cross-tissue” RNAseq dataset. Each tSNE visualization depicts the coexpression patterns of genes, with each color corresponding to the genes listed on the left, right, and bottom of the plot. The tissue name, as referenced on the Fly SCope website, is indicated in the upper left corner of the tSNE plot. Dashed lines denote the significant overlap of cell populations annotated by the respective genes. We have delineated the prominent cell populations using dashed lines on the tSNE plot to facilitate the readers' ability to discern clusters where multiple genes are co-expressed. This visualization approach was chosen to enhance the interpretability of the data. Coexpression between genes or annotated tissues is visually represented by differentially colored cell populations. For instance, yellow cells indicate the coexpression of a gene (or annotated tissue) with a red color and another gene (or annotated tissue) with a green color. Cyan cells signify coexpression between green and blue, purple cells for red and blue, and white cells for the coexpression of all three colors (red, green, and blue). Consistency in the tSNE plot visualization is preserved across all figures.

### Systematic analysis of cone cell enriched genes among wang *et al*'s microarray dataset

Different expressed genes and fold-changes in the head between single-housed and group-housed conditions from the *Drosophila melanogaster* were obtained by Wang's 2008 (Wang *et al*. 2008). Single-cell RNA sequencing (scRNA-seq) data from the *Drosophila melanogaster* were obtained from the Fly Cell Atlas website (https://doi.org/10.1126/science.abk2432). The UMIs data were retrieved, consisting of a grand total of 5,397 cells. The Seurat (v4.2.2) package (Hao *et al*. 2021) was utilized for data analysis. The “NormalizeData” function was utilized for the purpose of automated data normalization. Differential expression and fold-changes in expression in cone cells were calculated using the Seurat “FindMarkers” function.

### Statistical analysis

Statistical analysis of mating duration assay was described previously (Kim *et al*. 2012, 2013; Lee *et al*. 2023). More than 50 males (naïve, experienced, and single) were used for mating duration assay. Our experience suggests that the relative mating duration differences between naïve and experienced conditions and singly reared are always consistent; however, both absolute values and the magnitude of the difference in each strain can vary. So, we always include internal controls for each treatment as suggested by previous studies (Bretman, Westmancoat, *et al*. 2011). Therefore, statistical comparisons were made between groups that were naïvely reared, sexually experienced, and singly reared within each experiment. As the mating duration of males showed normal distribution (Kolmogorov–Smirnov tests, *P* > 0.05), we used two-sided Student's *t*-tests. The mean ± standard error (s.e.m) (**** = P < 0.0001, *** = P < 0.001, ** = P < 0.01, * = P < 0.05). All analysis was done in GraphPad (Prism). Individual tests and significance are detailed in figure legends. Besides traditional *t*-test for statistical analysis, we added estimation statistics for all MD assays and two groups comparing graphs. In short, “estimation statistics” is a simple framework that—while avoiding the pitfalls of significance testing—uses familiar statistical concepts: means, mean differences, and error bars. More importantly, it focuses on the effect size of one's experiment/intervention, as opposed to significance testing (Claridge-Chang and Assam 2016). In comparison to typical NHST plots, estimation graphics have the following five significant advantages such as (1) avoid false dichotomy, (2) display all observed values (3) visualize estimate precision (4) show mean difference distribution. And most importantly (5) by focusing attention on effect size, the difference diagram encourages quantitative reasoning about the system under study (Ho *et al*. 2019). Thus, we conducted a reanalysis of all our two group data sets using both standard *t*-tests and estimate statistics. In 2019, the Society for Neuroscience journal eNeuro instituted a policy recommending the use of estimation graphics as the preferred method for data presentation (Bernard 2021).

### Cone cell dissection and immunostaining

After 5 days of eclosion, the *Drosophila* cone cells were taken from adult flies by grasping the center of the posterior head capsule with forceps and carefully removing the mouthparts (proboscis), fat tissue, and tracheae. Then, one forceps was used to hold the dorsal head cuticle while the other was used to gently separate the eyes from the brain tissue by pulling sideways. After fixation, the lamina was removed by gently pulling it sideways with forceps. Any remaining cuticle was also removed to prevent it from folding onto the retina during mounting. The sample was then fixed in 4% formaldehyde at room temperature for 30 minutes, followed by three washes (5 minutes each) in 1% PBT. The sample was then blocked in 5% normal goat serum for 30 minutes. Subsequently, it was incubated overnight at 4°C with primary antibodies in 1% PBT, followed by the addition of fluorophore-conjugated secondary antibodies for one hour at room temperature. Finally, the cone cells were mounted on slides with an antifade mounting solution (Solarbio) for imaging purposes.

## Results

**Fig. 1.**
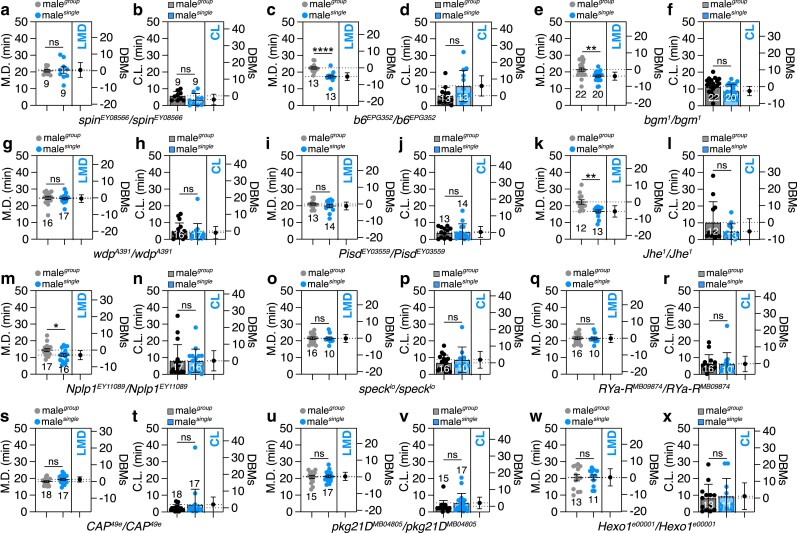
Single-housing upregulated genes are required for LMD but do not affect CL in male *Drosophila*. a–x) To check whether isolation-induced genes are required for LMD, MD, and CL of *spin*^*EY08566*^*/spin^EY08566^* (a and b), *b6*^*EPG352*^/*b6*^*EPG352*^ (c and d), *bgm*^*1*^*/bgm^1^* (e and f), *wdp*^*A391*^*/wdp^A391^* (g and h)*, Pisd^EY03559^*/*Pisd^EY03559^* (i and j), *Jhe*^*1*^*/Jhe^1^* (k and l), *Nplp1*^*EY11089*^/*Nplp1*^*EY11089*^ (m and n), *speck^lo^/speck^lo^* (o and p), *RYa-R*^*MB09874*^*/RYa-R^MB09874^* (q and r), *CAP*^*49e*^*/CAP^49e^* (s and t)*, pkg21D^MB04805^*/*pkg21D^MB04805^* (u and v), and *Hexo1*^*e00001*^*/Hexo1^e00001^* (w and x) males were tested in group and single conditions. In the mating duration (MD) assays, light gray data points denote males that were group-reared (or sexually naïve), whereas blue (or pink) data points signify males that were singly reared (or sexually experienced). The dot plots represent the MD of each male fly. The mean value and standard error are labeled within the dot plot (black lines). Asterisks represent significant differences, as revealed by the unpaired Student's *t*-test, and ns represents non-significant differences (*P < 0.05, **P < 0.01, ***P < 0.001, ****P < 0.0001).

In our investigation, we selected a subset of genes that were found to be upregulated in single-housing conditions based on previous microarray data (Wang *et al*. 2008). These genes were of interest due to their potential association with aggressive behavior in the context of social isolation and interaction. To assess their role in mating behaviors, we examined the phenotypic effects of mutants for these genes on both mating duration (or copulation duration) (MD) and copulation latency [or mating latency (CL)] (Supplementary Fig. 1a and b for schematic diagram of behavioral assay). In our LMD analysis, the presence of statistically significant differences (indicated by stars) between the mating durations of group-reared and singly reared flies signifies that the LMD behavior is functioning properly. Conversely, the absence of statistical significance (labeled as “ns” for non-significant) suggests that the LMD behavior is impaired or disrupted. This same approach can be used to interpret the CL patterns when comparing group-reared vs singly reared flies (e.g. compare Fig. 1a–d).

In the context of *Drosophila* reproductive strategies, it is established that male activity and female receptivity are pivotal to the success of mating events (Bastock 1956). The intensity of a male's mating effort is commonly assessed by proxy through MD, which is inversely associated with the temporal window for female remating; a longer MD typically results in an extended interval before a female will reengage in mating. Males that engage in extended copulation periods effectively diminish the competitive potential of rival sperm at a relatively low energetic cost. Furthermore, there is a direct correlation between the length of mating and the male's investment in sperm (Bretman *et al*. 2009; Price *et al*. 2012; Bretman, Westmancoat, and Chapman 2013; Bretman, Westmancoat, Gage, *et al*. 2013; Rouse and Bretman 2016; Dore *et al*. 2021).

The significance of male CL—the temporal interval from male encounter to copulation initiation—is paramount for reproductive fitness in *Drosophila*. A reduced CL enhances a male's probability of securing a mating opportunity before competitors, thereby potentially increasing his reproductive success. Moreover, efficient mating, characterized by swift initiation, can lead to a greater number of progeny, thereby amplifying the male’s genetic contribution to subsequent generations (Van den Berg 1986; Krishna and Hegde 2003; Pavković-Lučić and Kekić 2009; Singh and Singh 2014; Fricke *et al*. 2020).

The decision to include CL in our analysis was informed by the existing literature, which reports that CL remains consistent despite variations in MD between group-housed and single-housed individuals (Kim *et al*. 2012). Through the comparative analysis of two distinct timing behaviors, both of which are associated with the reproductive fitness of male *Drosophila*, we propose a more precise evaluation of the impact of genetic variables on LMD behavior.

### The predominant subset of genes identified through the social interaction microarray analysis exhibits a significant association with LMD behavior

Drawing from the cohort of genes upregulated in singly reared males as reported by Wang *et al*. (Wang *et al*. 2008), we focused on those genes that demonstrated the most significant differential expression ratio between group-reared (G) and singly reared (S) males (G:S ratio), ensuring that the selected genes corresponded to strains with available homozygous viable mutants. This selection criterion enabled us to investigate the behavioral impacts of these genes without confounding effects from developmental lethality. In instances where homozygous mutants were lethal, we elected to test hemizygous mutants. Of the 114 genes identified as upregulated in Wang *et al*.'s microarray analysis, we selected 23 genes with viable homozygous mutants and an additional 4 genes for which the mutation results in homozygous lethality.

Our findings revealed that the majority of mutants exhibited significant alterations in LMD behavior. However, their CL remained largely unchanged between group and single condition (Fig. 1 and Supplementary Fig. 1a–d). This suggests that the genes under investigation primarily influence the MD rather than the CL.

Notably, mutants for the genes *b6*, *bubblegum* (*bgm)*, *Juvenile hormone esterase (Jhe)*, and *Neuropeptide-like precursor 1 (Nplp1)* demonstrated normal LMD behavior and CL (Fig. 1c, e, k, and m). These results imply that these specific genes, previously implicated in aggression, do not have a significant impact on mating behavior. This distinction underscores the complex relationship between aggressive behavior and reproductive strategies, suggesting that while certain genes may be involved in both, they do not necessarily influence mating duration or latency in *Drosophila*.

To investigate the transcriptional profiles of genes of interest, we utilized the single-cell RNA sequencing dataset available from the recently established fly SCope platform (Li *et al*. 2022). For the representation of microarray data, we selected the *Cyp6a20* gene as a positive control (Wang *et al*. 2008), and the *doublesex* (*dsx*) gene was employed as a marker for male-specific cells. The *dsx* gene in flies plays a critical role in determining male-specific behaviors and physiology by regulating the expression of genes that are involved in the development of male traits (Yamamoto *et al*. 1998; Kunte *et al*. 2014). We utilized a tSNE plot to illustrate the gene expression patterns within adult fly head tissue. Each dot on the plot corresponds to a single cell, with colors indicating the presence of specific genes: red for one gene, green for another, and blue for a third. In the absence of expression for any of the three genes within a cell, the corresponding cell will be displayed in a dark gray color. When a cell expresses only one gene, the dot retains its original color. However, when two or more genes are co-expressed within the same cell, the dot color changes to a blend, such as yellow for red and green, cyan for green and blue, purple for red and blue, or white/light gray for all three genes co-expressed together. We have identified and marked key cell clusters on the tSNE plot that express both our genes of interest and specific marker genes using red dotted lines. It's important to note that the presence of white cells, indicating coexpression of all three genes, does not necessarily imply functional interaction among them. Even if mutant genes co-express with *Cyp6a20* and *dsx*, the mutants can still exhibit normal LMD behavior. The purpose of the tSNE plot was to demonstrate whether the gene expression patterns of our screened genes are closely related to those of previously characterized marker genes.

Notably, genes implicated in the modulation of LMD when mutated exhibited significant coexpression with both *dsx* and *Cyp6a20* within discrete cell populations of the adult fly head (Supplementary Fig. 1g–s). Conversely, genes unrelated to LMD, such as *b6*, *bgm*, *Jhe*, and *Nplp1*, demonstrated minimal overlap in expression with *dsx* or *Cyp6a20* (Supplementary Fig. 1j, k, n, and o). We did not investigate the colocalization of the identified genes with *fruitless* (*fru*), another pivotal regulator of male-specific development (Siwicki and Kravitz 2009), due to the extensive expression of *fru* in the fly head single-cell RNA sequencing dataset, which obfuscated the identification of significant coexpression patterns (Supplementary Fig. 1t). In summary, these findings suggest that the gene expression profiles of these specific cell populations are pivotal in the manifestation of LMD behavior.

**Fig. 2.**
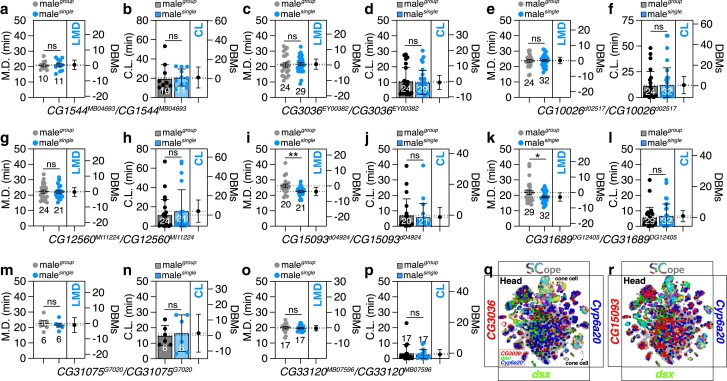
Male–male interaction-regulated genes are required for LMD but do not affect CL in male *Drosophila*. a–p) To screen genes linked to male social interactions required for LMD, MD and CL of *CG1544*^*MB04693*^/*CG1544*^*MB04693*^ (a and b), *CG3036*^*EY00382*^/*CG3036*^*EY00382*^ (c and d), *CG10026*^*d02517*^/*CG10026*^*d02517*^ (e and f), *CG12560*^*MI11224*^*/CG12560^MI11224^* (g and h), *CG1509*3*^d04924^*/*CG1509*3*^d04924^* (i and j), *CG31689*^*DG12405*^/*CG31689*^*DG12405*^ (k and l), *CG31075*^*G7020*^/*CG31075*^*G7020*^ (m and n), and *CG33120*^*MB07596*^/*CG33120*^*MB07596*^ (o and p) males were tested in group and single conditions. *, *P* < 0.05; **, *P* < 0.01 (unpaired *t*-test). q–r) *CG3036* predominantly overlaps *dsx* and *Cyp6a20* in cone cells within the head, as opposed to *CG15093*. *t*-distributed stochastic neighbor embedding (tSNE) representation of scRNA-seq datasets from individual cells of the *D. melanogaster*—generated as part of the Fly Cell Atlas (10× stringent dataset) within the head colored for gene expression: *CG3036* (q) and *CG15093* (r) in red, *dsx* in green, and *Cyp6a20* in blue. Each tSNE visualization depicts the coexpression patterns of genes, with each color corresponding to the genes listed on the left, right, and bottom of the plot. The tissue name, as referenced on the Fly SCope website is indicated in the upper left corner of the tSNE plot.

In *Drosophila*, gene nomenclature often reflects the gene's function, observed mutant phenotype, or other pertinent attributes. Ideally, *Drosophila* gene names should be succinct, distinctive, and free from offensive connotations (Roberts 2006; Attrill *et al*. 2016). Following our initial mini-genetic screening focused on genes with established names, we elected to expand our screening to include genes lacking naming, which may not have had their functions delineated. Our objective with these unnamed genes was to identify those specifically implicated in male–male interaction phenotypes. Consequently, to corroborate the microarray data and to investigate the functional significance of genes of unknown function, we selected a cohort of these genes for in-depth analysis. Of the ten genes tested, mutants for seven genes exhibited disrupted LMD phenotypes without concomitant defects in CL (Fig. 2a–p and Supplementary Fig. 2a–d). This observation indicates that genes whose expression is modulated by male–male social interactions are strongly correlated with the manifestation of LMD behavior.

Consistent with the findings from known genes, the genes associated with LMD demonstrated significant overlap in expression with both *dsx* and *Cyp6a20* (Fig. 2q and r and Supplementary Fig. 2e–l). These results implicate genetic factors that are dynamically regulated by male–male interactions as critical components in the generation of LMD behaviors, specifically within certain cell populations of the adult fly head, without influencing CL.

### Cone cells represent a principal target tissue for genetic modulation of LMD behavior

**Fig. 3.**
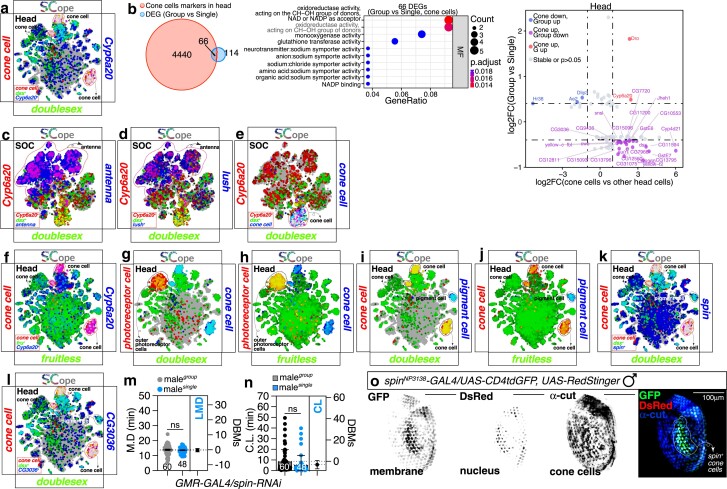
Differentially expressed genes (DEGs) in cone cells of *Drosophila melanogaster* between Group and Single conditions. a) *Cyp6a20* predominantly overlaps *dsx* in cone cells within the head. b) (left) A Venn diagram depicting the DEGs observed between single-housed and group-housed flies, as well as markers in cone cells among head cells. (middle) GO pathway enrichment analysis. The dot plot shows the GO terms associated with the 66 DEGs identified for molecular functions. The size of each dot represents the gene count enriched in the pathway, and the color indicates the significance of the pathway enrichment. (right) Volcano plot illustrating changes in the expression of cone cell marker genes under single-housed vs group-housed conditions. The *x*-axis shows fold-changes in gene expression between cone cells and other head cells. The *y*-axis shows fold-changes in gene expression between single-housed and group-housed. c-e) *Cyp6a20*, *lush* and cone cells, co-express with *dsx* within the head. tSNE colors are based on cell types or gene expression: *Cyp6a20* in red, *dsx* in green, and *antenna* (c) and *lush* (d) and *cone cells* (e) in blue. f) *fru* is significantly expressed in cone cells within the head. tSNE colors are based on cell types or gene expression: cone cells in red, *fru* (f) in green, and *Cyp6a20* in blue. g and h) Outer photoreceptor cells express *dsx* less than *fru*, unlike cone cells within the head. tSNE colors are based on cell types or gene expression: outer photoreceptor cells in red, *dsx* (g) and *fru* (h) in green, and cone cells in blue. i and j) Both *fru* and *dsx* are strongly expressed in pigment cells within the head. tSNE colors are based on cell types or gene expression: cone cells in red, *dsx* (i) and *fru* (j) in green, and pigment cells in blue. k and l) LMD-related genes, *spin* and *CG3036*, co-express with *dsx* in cone cells within the head. tSNE colors are based on cell types or gene expression: cone cells in red, *dsx* in green, *spin* (k) and *CG3036* (l) in blue. m and n) MD and CL assay for GAL4-mediated knockdown of spin *via spin-RNAi* using the *GMR-GAL4* driver. ns represents non-significant difference (unpaired *t*-test). o) The expression pattern of *spin*-*GAL4* in adult cone cells. Cone cells of male flies expressing *GAL4*^*spin*^ together with *UAS-mCD8GFP, UAS-RedSting* were immunostained with anti-cut (blue) antibody. Scale bars represent 100 μm. Dashed circles indicate the region of interest.

Utilizing the RNA sequencing dataset from the fly atlas (http://flycellatlas.org) (Scheffer *et al*. 2020; Li *et al*. 2022), we discerned that the cell populations exhibiting the highest expression of genes identified through our screening, which also show significant overlap with *dsx* and *Cyp6a20* (Fig. 2q and r) and correspond to cone cells (Fig. 3a). To systematically determine which tissues the gene expression changes occur in male’s social context, we reanalyzed Wang *et al*.’s screening data (Wang *et al*. 2008) along with FCA scRNA-seq data (Li *et al*. 2022) to assess differentially expressed genes (DEGs) in *Drosophila melanogaster* reared in single-housed vs group-housed settings. We focused on DEGs that might serve as markers for cone cells. Out of 114 DEGs described in a previous study, 66 genes of that were specifically altered in cone cells (left Venn diagram of Fig. 3b), indicating a significant change in cone cells between the two conditions. Gene Ontology (GO) analysis of these 66 DEGs revealed an association with oxidoreductase activity (middle panel of Fig. 3b). Notably, under single-housed conditions, most of these genes were upregulated. In contrast, *hormone receptor-like in 38 (Hr38)*, which encodes a protein involved in glycogen storage, and *insulin-like peptide 3 (Dilp3)* were upregulated under group-housed conditions (right volcano plot of Fig. 3b). These results suggest metabolic changes in cone cells. Overall, our findings highlight significant alterations in cone cell activity between single-housed and group-housed conditions, with increased expression of oxidoreductase activity genes under single-housed conditions and decreased metabolic processes under group-housed conditions.

Wang *et al*. previously identified that *Cyp6a20* is expressed in a specific subset of olfactory sensory organs in association with the odorant binding protein (OBP) LUSH, which characterizes a subpopulation of non-neuronal support cells (Kim *et al*. 1998; Smith 2007; Wang *et al*. 2008). We confirmed *Cyp6a20* expression in antenna tissue using fly atlas RNAseq data (Li *et al*. 2022) (Fig. 3c, indicated by purple cells), where *lush* is highly expressed (Fig. 3d, also indicated by purple cells). However, the antennae expressing *Cyp6a20* do not overlap with those expressing the *dsx* gene (Fig. 3c and d, indicated by white cells). Interestingly, among the *Cyp6a20*-positive cells in the sensory organ cell (SOC) population, the majority of *dsx*-positive cells are labeled as cone cells. These findings suggest that Cyp6a20 may have distinct functions in olfactory and visual sensory organs, particularly highlighting the possibility that the sex-specific function of Cyp6a20 is enriched in the cone cell population that coexpresses the *dsx* gene.

In *D. melanogaster*, cone cells are responsible for the secretion of the lens material that is essential for the optically correct functioning of the compound eye (Cagan and Ready 1989). Surprisingly, our data showed that the *fruitless* gene is not significantly expressed in cone cells (Fig. 3f, indicated by white cells), even though it is known to play a vital role in male-specific behaviors and is necessary for the brain circuitry that controls courtship in *D. melanogaster* (Yamamoto and Koganezawa 2013). Outer photoreceptor cells, which are neuronal in nature, showed higher levels of *fru* expression compared to *dsx*, in sharp contrast to cone cells (Fig. 3g and h). In contrast, pigment cells exhibited strong expression of both *fru* and *dsx* (Fig. 3i and j). Previous studies have shown that the *fru* gene, which is mainly expressed in the nervous system, is necessary for male courtship behaviors. On the other hand, the *dsx* gene, which has a wider expression pattern, plays a crucial role in coordinating sexual differentiation in both neural and non-neural tissues (Wasserman 2000; Stockinger *et al*. 2005; Siwicki and Kravitz 2009; Rideout *et al*. 2010; Dauwalder 2011; Yamamoto and Koganezawa 2013). Since cone cells, which are non-neuronal and provide structural support to the compound eye, do not play a role in the neural processes of sexual behavior, they mainly express *dsx* for the differentiation of physical sex characteristics rather than *fru*.

Genes identified from the screening for LMD, such as *spin* and *CG3036*, exhibited a strong pattern of coexpression with *dsx* in cone cells (Fig. 3k–i, indicated by white cells). The observed coexpression pattern implies that *dsx*-positive cone cells serve as a critical site for the modulation of gene expression associated with aggressive behavior in response to male–male interactions or social isolation (Wang *et al*. 2008). To substantiate the hypothesis that cone cells represent the minimal cellular cohort responsible for the social interaction-dependent gene expression changes underlying LMD, we examined a variety of tissues annotated in the fly SCope database. We found that cone cells, identified within the SOC and epithelial cell annotation, were the predominant cell type expressing *dsx* along with *Cyp6a20* (Supplementary Fig. 2m–q), indicating their pivotal role in mediating the LMD.

### Diverse cone cell subsets within the compound eye may contribute variably to the modulation of LMD behavior

To validate the role of identified genes in the cone cell compartment, we employed an RNA interference (RNAi) approach to knock down the spin gene expression specifically in the compound eye of *Drosophila melanogaster*, utilizing the GMR-GAL4 driver that selectively targets this tissue (Escobedo *et al*. 2021). Immunostaining with the cone cell-specific marker anti-cut antibody confirmed the specificity of GMR-GAL4 for cone cells in both sexes of the compound eye (Blochlinger *et al*. 1988) (Supplementary Fig. 3a and b). The targeted knockdown of spin in the compound eye resulted in the disruption of LMD behavior without compromising CL (Fig. 3m and n), indicating that spin’s function within the cone cell population is pivotal for the generation of LMD but not for the maintenance of normal CL. Employing an enhancer trap line with a spin-GAL4 driver (Smith *et al*. 2015), we observed that spin is expressed in a discrete domain within the male compound eye (Fig. 3o). The cone cell marker, nuclear-localized CUT protein, showed colocalization with UAS-RedStinger signals, which were confined to the nucleus (Fig. 3o, white dashed circle) (Blochlinger *et al*. 1988; Pickup *et al*. 2009), thereby confirming that spin-expressing cells constitute a distinct cone cell population within the compound eye.

**Fig. 4.**
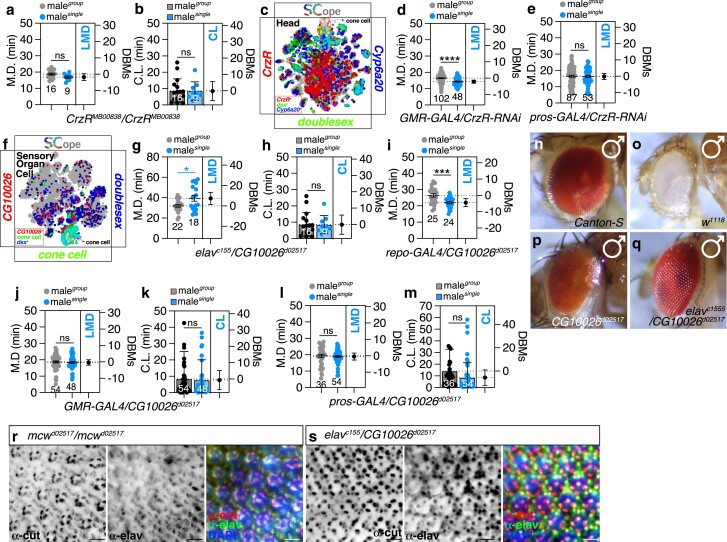
Different cone cell subsets in the eyes and the metabolic gene *CG10026* modulate LMD behavior. a and b) MD and CL of *CrzR*^MB00838^/*CrzR*^MB00838^ males in LMD condition. ns represents non-significant difference (unpaired *t*-test). c) *CrzR* is expressed at high levels in *dsx*-positive cone cell populations. tSNE colors are based on cell types or gene expression: *CrzR* in red, *dsx* in green, and *Cyp6a20* in blue. d and e) LMD assay for GAL4-mediated knockdown of CrzR *via CrzR-RNAi* using the *GMR-GAL4* and *pros-GAL4* driver. ns represents non-significant difference (unpaired *t*-test). f) *CG10026* is expressed in cone cells that overlaps *dsx* within the sensory organ cells. tSNE plots colored by cell types or gene expression: *CG10026* in red, cone cells in green, and *dsx* in blue. g and h) MD and CL for GAL4-mediated overexpression of CG10026 *via CG10026*^*d02517*^ using *elav*^*c155*^ in LMD condition. *, *P* < 0.05; ***, *P* < 0.001 (unpaired *t*-test). i) LMD assay for GAL4-mediated overexpression of CG10026 *via CG10026*^*d02517*^ using *repo-GAL4*. ***, *P* < 0.001 (unpaired *t*-test). j-m) MD and CL for GAL4-mediated overexpression of CG10026 *via CG10026*^*d02517*^ using *GMR-GAL4* and *pros-GAL4* in LMD condition. *, *P* < 0.05; ***, *P* < 0.001 (unpaired *t*-test). n-q) Identification and characterization of different genotypes. Canton-S (CS) fly showing normal eye phenotype (n and o). *CG10026*^*d02517*^and overexpression of CG10026 *via CG10026*^*d02517*^ using *elav*^*c155*^shows normal eye phenotype (p and q). r and s) Features of *mcw^d02517^* and *CG10026 via CG10026^d02517^* using *elav*^*c155*^ adult male eyes. Cone cells of male flies *mcw^d02517^* and *CG10026 via CG10026^d02517^* using *elav*^*c155*^were immunostained with anti-cut (red) and anti-elav(green) antibodies. Scale bars represent 100 μm. Dashed circles indicate the region of interest.

One of the genes whose expression was highly changed by single-housing was Crz receptor (CrzR) and CrzR mutant showed disrupted LMD but normal CL (Fig. 4a and b). The *Drosophila melanogaster* Crz-CrzR system is a hormonal signaling pathway that plays a key role in regulating stress responses and physiological homeostasis in the fruit fly. In addition to its role in stress responses, the Crz-CrzR system has also been implicated in regulating sleep, aggression, and other behaviors in *Drosophila* (Johnson *et al*. 2005; Veenstra 2009; Gospocic *et al*. 2017; Zandawala *et al*. 2021). At the core of this system is the Crz, a peptide hormone that functions as a neuromodulator and stress hormone. Crz is produced by specific neurons in the brain and is released into the circulation, where it binds to its receptor, CrzR, which is expressed in various target tissues throughout the fly's body (Kim *et al*. 2004; Johnson *et al*. 2005).

Notably, the Crz-CrzR system in *Drosophila melanogaster* has been identified as a critical regulator of both the mating duration (Wong *et al*. 2019) and the process of sperm transfer, functioning within sexually dimorphic neural circuits that are modulated by the *doublesex (dsx)* gene (Zhao *et al*. 2010; Tayler *et al*. 2012). Our findings revealed that the CrzR is expressed at high levels within *dsx*-positive cone cell populations located at the center top region of tSNE plot of head tissue (Fig. 4c). Intriguingly, the RNAi-mediated knockdown of CrzR using the GMR-GAL4 driver did not alter LMD behavior (Fig. 4d), in contrast to the disruption of LMD observed with *spin-RNAi* under the same driver (Fig. 3m and Supplementary Fig. 3h and i for genetic control). Utilizing the fly SCope tSNE plot, we discerned that CrzR colocalizes with Cyp6a20 within a specific cone cell population situated at the central superior region of the head tSNE plot (compare Fig. 4c with Supplementary Fig. 3c). However, the glass (gl) gene, the promoter region of which was employed for the cloning of GMR-GAL4 (Ray and Lakhotia 2015), does not colocalize with this cone cell population (Supplementary Fig. 3c, compare yellow vs cyan dashed circles), suggesting that GMR-GAL4 may not target a particular subset of cone cells where CrzR functions to modulate LMD behavior.

To delineate the functional role of CrzR expression in cone cells for the induction of LMD behavior, we sought and applied the pros-GAL4 driver, previously reported to be expressed in cone cells (Kumar *et al*. 2015), and observed that RNAi-mediated reduction of CrzR in this cone cell subtype led to the impairment of LMD (Fig. 4e and Supplementary Fig. 4l and m for genetic control). Notably, we confirmed that the *prospero* (*pros*) gene, a cone cell marker, is expressed in a confined area of the cone cell population located at the inferior right quadrant of the head tSNE plot (Supplementary Fig. 3d, indicated by cyan dashed circles). Furthermore, we found that the *pros*-positive cone cell population exhibits high expression in cells that also express *dsx*, as annotated within the epithelial cell category (Supplementary Fig. 3e, indicated by white dashed circles). The inner layer of the compound eye cone cells was effectively labeled by pros-GAL4 in conjunction with the anti-cut antibody (Supplementary Fig. 3f), whereas only a subset of cone cells in the outer layer was labeled by the pros-GAL4 driver (Supplementary Fig. 3g, indicated by yellow dashed circles). These findings collectively indicate that CrzR expression in pros-positive subtype of cone cells is a critical determinant in the regulation of LMD behavior.

### The cell-type-specific expression of metabolic genes within cone cells is crucial for the induction of LMD behavior

Recent studies demonstrated that beta-alanine, a key molecule in both glucose metabolism and photoreceptor neurotransmitter recycling, is highly expressed in the *Drosophila* pseudocone (Borycz *et al*. 2012). Together, these data suggest that cone cells could provide structural and functional support for photoreceptors in the fly retina, similar to Muller glia in the vertebrate retina (Fischer and Bongini 2010). Consistent with this, cone cells are the first non-neuronal cells to form after neurogenesis, like glia in other parts of the nervous system. It has been known that the bifunctional role of cone cells in the insect compound eye is not only necessary for lens formation as previously described but also for sustaining the functionality of photoreceptor cells as resident glia/support cells. Thus, metabolic genes expressed in cone cells are critical-to-modulate fly vision.

Microarray analysis of gene expression patterns in socially isolated male *Drosophila melanogaster* revealed a significant enrichment of metabolic genes among those that were altered in expression. Specifically, the *Cyp6a20* gene, known to play a critical role in the modulation of aggressive behaviors during social interactions, was identified as a prominent metabolic regulator within this subset of genes (Wang *et al*. 2008).

To comprehensively investigate the impact of the metabolic gene expression on LMD behavior in cone cell populations, we specifically targeted the *CG10026* gene for functional validation (Fig. 2e and f). The *CG10026* gene is a nuclear-encoded protein that is expressed across diverse tissues and cell types in fruit flies, including the membranes of adult fly heads. This gene and its encoded proteins are associated with a range of functions, such as binding to phosphatidylinositol bisphosphate and are predicted to participate in intermembrane lipid transfer (Gramates *et al*. 2022; Jenkins *et al*. 2022). The CG10026 mutant trans-heterozygote over wild-type strain showed normal LMD behavior (Supplementary Fig. 4a), suggesting that the CG10026 mutant phenotype is recessive. Expression of CG10026 is enriched in specific cell populations among *dsx*-positive cone cells, which are annotated as SOC (Fig. 4f) rather than epithelial cells (Supplementary Fig. 4b). The tSNE plot analysis indicates that the function of CG10026 is likely associated with neural progenitor cells, rather than epithelial origins.

Given that the *CG10026*^*d02517*^ mutant contains a UAS regulatory sequence suitable for misexpression screening (Thibault *et al*. 2004), we employed *elav*^*c155*^ to overexpress CG10026 in neural progenitor cells or *repo-GAL4* in glial progenitor cells. Our findings indicate that only overexpression of CG10026 in *elav*-positive, but not *repo*-positive, cells disrupt LMD behavior (Fig. 4g–i). This suggests that the CG10026-expressing cone cell population, which highly expresses *elav* but not *repo* (Supplementary Fig. 4c and d), may be critical in modulating mating duration, with the expression level of CG10026 in *elav*-positive cells being a key component for LMD. The gene *CG10026* has been named “*Macewindu (mcw)*” after the Jedi Master who failed to detect the Sith Lord Palpatine, paralleling the mutant's inability to detect rivals and the absence of LMD behavior (MacDubhghaill 2019). The ectopic expression of *mcw* in specific subsets of cone cells, utilizing the GMR-GAL4 and pros-GAL4 drivers, resulted in the impairment of LMD behavior without altering CL (Fig. 4j–m). This observation implies that *mcw* plays a role in mating behavior across the majority of cone cell populations targeted by these GAL4 drivers. No significant differences in compound eye morphology were detected between *mcw* mutant or neuronal overexpression lines compared to wild-type strains (Fig. 4n–q), indicating that both the absence and overexpression of *mcw* do not impact the structural integrity of the compound eye.

### The integrity of cone cell structure is intrinsically linked to the manifestation of LMD behavior

To elucidate the role of *mcw* in cone cell functionality, we first conducted a detailed analysis of the cone cell layer in both mutant and overexpressed lines. The cone cells in the wild-type Canton-S strain exhibited a structured morphology, as indicated by anti-cut antibody and anti-elav staining, which delineated neuronal progenitors within the cone cell layer (Supplementary Fig. 4e). In contrast, the *w^1118^* strain displayed a degenerative cone cell phenotype relative to the normal neuronal architecture (Supplementary Fig. 4f). As previously reported, *w^1118^* males exhibit aberrant LMD behavior (Kim *et al*. 2012), and it has been documented that the *w^1118^* mutant undergoes retinal degeneration (Ferreiro *et al*. 2018). The *white* gene's influence on courtship behavior has been a subject of frequent discussion (Krstic *et al*. 2013), with misexpression leading to male–male courtship (Zhang and Odenwald 1995) and the gene itself being implicated in the success of copulation (Xiao *et al*. 2017). The *white* gene, initially identified in 1910 by Thomas Hunt Morgan (Morgan 1910), encodes a subunit of an ATP-binding cassette (ABC) transporter involved in pigment granule loading and deposition in various tissues, including the compound eyes and the nervous system (Ewart and Howells 1998; Borycz *et al*. 2008; Xiao and Robertson 2016). Therefore, we propose that the degeneration of cone cells in the *w^1118^* mutant may impair LMD behavior by disrupting proper visual recognition of competitors.

The gene *Crystallin* (*Crys*), which encodes a glycoprotein contributing to the formation of the corneal lens in the fly’s compound eye, has not been extensively studied. However, it is recognized as a major component of the corneal lens cuticular proteins, which include Crys, retinin, Cpr66D, and Cpr72Ec. It has been confirmed that Crys and retinin are expressed in the primary corneagenous cells—cone cells and primary pigment cells (Stahl *et al*. 2017). Crys also plays a role in resistance to oral infection with S. aureus in adult *Drosophila* (Hori *et al*. 2018). We observed that the cone cell structure in *Crys* mutants was severely malformed (Supplementary Fig. 4g) and that LMD behavior was disrupted in these mutant males, without affecting CL (Supplementary Fig. 4h and i). Knockdown of *retinin*, another key corneal lens protein, also resulted in the impairment of LMD behavior without altering CL (Supplementary Fig. 4j and k), indicating that the corneal lens-supporting functions of Crys and retinin within cone cells are essential for the generation of LMD behavior. The *retinin* gene encodes a corneal-specific protein (Kim *et al*. 2008), and RNAi knockdown of *retinin* with GMR-GAL4 reduced the nipple height of the compound eye (Kryuchkov *et al*. 2020). These findings collectively suggest a critical association between the structural integrity of cone cells and the execution of LMD behavior.

Remarkably, the structural morphology of cone cells in *mcw* mutant males appears to be intact (Fig. 4r). However, upon misexpression of *mcw* using the *elav*^*c155*^ driver in neural progenitor cells, we observed that the cone cell structure became hypertrophic and exhibited enhanced structural regularity (Fig. 4s). We hypothesize that this phenotype may correlate with the reversed LMD phenotype observed in Fig. 4g, where singly reared males exhibit extended mating durations compared to group-reared males. These findings suggest that the overexpression of *mcw* in the compound eye could result in an elongation of the mating duration in singly reared males. Consequently, we propose that the level of mcw protein is a critical factor in the maintenance of cone cell function.

### The CYP gene family specifically expressed in cone cells is implicated in the modulation of LMD behavior

The original Wang *et al*.’s study delineated the differential expression of genes between solitary and group-housed male *Drosophila*, with a particular emphasis on the *Cyp6a20* gene (Wang *et al*. 2008). Cyp6a20 belongs to the Cytochrome P450 (CYP) family, a collective of enzymes with a broad and multifunctional role in insect metabolism. These enzymes are pivotal in the detoxification of xenobiotics, the synthesis of hormones, and the regulation of various physiological processes (Scott and Wen 2001; Dang *et al*. 2017; Denecke *et al*. 2017). The precise mechanisms by which Cyp6a20 influences aggression are yet to be fully elucidated; however, it is postulated that this gene is involved in the metabolism of signaling molecules and the regulation of neurotransmitter levels in the brain.

**Fig. 5.**
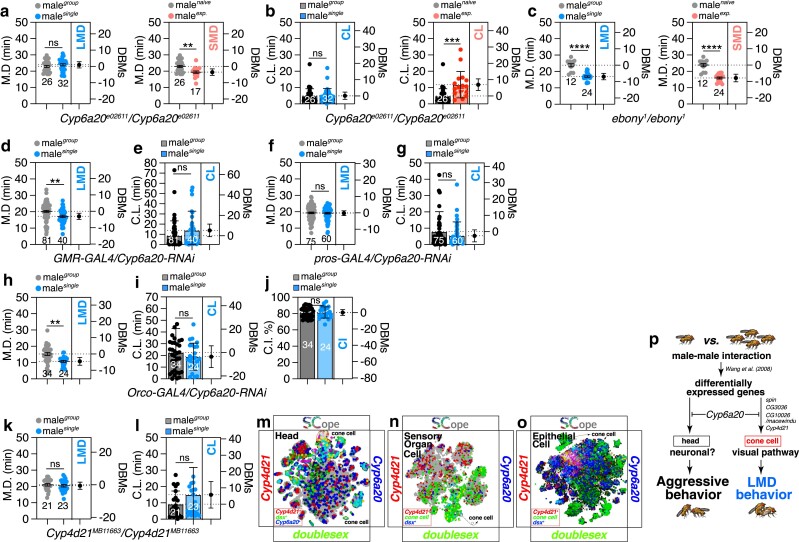
The metabolic genes (*Cyp6a20* and *Cyp4d21*) in cone cells are crucial for inducing LMD behavior. a and b) MD and CL of *Cyp6a20*^*e02611*^/*Cyp6a20*^*e02611*^ males in LMD and SMD conditions. **, *P* < 0.01, ***, *P* < 0.001 (Student's *t*-test). c) LMD and SMD assays of *ebony*^1^/*ebony*^1^ males. ****, *P* < 0.0001. d-g) MD and CL assays of GAL4-mediated knockdown of Cyp6a20 *via Cyp6a20-RNAi* using the *GMR-GAL4* and *pros-GAL4* driver. ns represents non-significant difference (unpaired *t*-test). h-j) MD, CL and CI assays of GAL4-mediated knockdown of Cyp6a20 *via Cyp6a20-RNAi* using the *Orco-GAL4* driver. ns represents non-significant difference (unpaired *t*-test). k and l) MD and CL assays of *Cyp4d21*^*MB11663*^*/Cyp4d21^MB11663^* males in LMD condition. ns represents non-significant difference (unpaired *t*-test). m-o) tSNE plots colored by cell types or gene *Cyp4d21* (red) and *Cyp6a20/dsx* (blue/green) within head (m), the sensory organ cells (n), and epithelial cells (o). p) Summary of this study showing cone cells are crucial for modulating LMD behavior.

To ascertain whether Cyp6a20 also impacts LMD in addition to aggression, we analyzed Cyp6a20 mutants and observed that LMD was disrupted, whereas shorter-mating duration (SMD) remained unaffected (Fig. 5a). The mating duration in male fruit flies, *Drosophila melanogaster*, serves as a valuable model for studying interval timing behaviors (Huang *et al*. 2024; Zhang *et al*. 2024). In *Drosophila*, two distinct interval timing behaviors related to mating duration have been identified including LMD, which occurs when males are in the presence of competitors and results in an extended copulation time (Bretman *et al*. 2009; Kim *et al*. 2012, 2013), and SMD, which is characterized by a reduction in mating duration and is observed in sexually experienced males (Lee *et al*. 2023). Furthermore, Cyp6a20 mutants displayed normal CL (Fig. 5b), and the heterozygote over wild-type strain exhibited normal LMD and SMD (Supplementary Fig. 5a), suggesting that the gene's function is specifically associated with rivalry-induced behaviors such as aggression and LMD rather than with behaviors dependent on female experience, such as SMD or delayed CL (Fig. 5b) in sexually experienced males (Lee *et al*. 2023). LMD behavior is critically dependent on the visual processing pathway (Kim *et al*. 2012), whereas SMD behavior is primarily dependent on the gustatory processing pathway (Lee *et al*. 2023). Consequently, the specific focus of the Cyp6a20 mutant phenotype on LMD behavior suggests that the function of Cyp6a20 within the visual system is intricately involved in the regulation of LMD behavior.

In our previous studies, we demonstrated that *white* mutants, characterized by visual deficits and increased aggression compared to wild-type males, exhibit disrupted LMD behavior (Edwards *et al*. 2009; Kim *et al*. 2012). To further investigate the relationship between genes linked to aggression and mating duration, we employed *ebony* gene mutant, which is known to display elevated aggression (Pantalia *et al*. 2023). The ebony gene encodes a protein that functions to conjugate beta-alanine to biogenic amines such as dopamine and histamine, thereby regulating the levels of free biogenic amines. For instance, it controls the availability of dopamine in cuticle formation and histamine in visual signal transduction within the eye. Moreover, the ebony gene is involved in behavioral rhythmicity. Notably, *ebony*^*1*^ mutants exhibited normal LMD (Fig. 5c) and SMD, but exhibited defects in CL (Supplementary Fig. 5b), indicating that not all genes associated with aggression have an impact on mating duration.

RNA interference-mediated knockdown of *Cyp6a20* in cone cells using the GMR-GAL4 driver did not alter LMD or CL (Fig. 5d and e and Supplementary Fig. 5e for genetic control). Conversely, knockdown of *Cyp6a20* in cone cells with the pros-GAL4 driver completely abolished LMD behavior without affecting CL (Fig. 5f and g), indicating that Cyp6a20 functions in a specific subset of cone cells, similar to CrzR, to regulate LMD behavior (Fig. 4d). To investigate the role of Cyp6a20 in the olfactory pathway and its influence on LMD, we utilized the Orco-GAL4 driver to knockdown Cyp6a20 in the majority of olfactory neurons. Surprisingly, the knockdown of Cyp6a20 in olfactory neurons did not impact LMD, CL, or CI behaviors (Fig. 5h-j and Supplementary Fig. 5f for genetic control). This suggests that the function of Cyp6a20 in the antenna is not directly linked to LMD behavior, contrary to the suggestion by Wang *et al*. that Cyp6a20 may be involved in aggression-related processes in the antenna (Wang *et al*. 2008). This result aligns with our previous findings indicating that the olfactory pathway does not contribute to the generation of LMD behavior (Kim *et al*. 2012). In summary, our findings indicate that Cyp6a20 likely has diverse functions in the antenna and compound eye, which collectively mediate aggression or mating behaviors in response to different social contexts.

CYP is a typical P450 enzyme family that participate in the metabolic processing of a diverse array of signaling molecules, including hormones, vitamins, and oxylipins (Tijet *et al*. 2001). Among the CYP family of genes, *Cyp4d21/Sxe1(Sex-specific enzyme 1)* was found to be differentially expressed in contexts involving male–male social interactions, as well as *Cyp6a20* (Wang *et al*. 2008). *Cyp4d21* mutants exhibited disrupted LMD behavior, while control genetic crosses with wild-type flies demonstrated normal LMD and CL (Fig. 5k and l and Supplementary Fig. 5c and d), indicating that *Cyp4d21* is specifically involved in LMD, not CL. *Cyp4d21* co-expresses with *Cyp6a20* and *dsx* in cone cells, which are annotated as head (Fig. 5m, indicated as white circles), sensory organ (Fig. 5n, indicated as white circles), and epithelial (Fig. 5o, indicated as white circles) tissues. These findings suggest that Cyp4d21 functions in cone cells to support LMD behavior through sex-specific mechanisms.

Previous research characterized Cyp4d21 as exhibiting differential expression in the heads of male and female *Drosophila*, with its role categorized as contributing to sex-specific physiological processes, particularly in adipose tissue (Fujii and Amrein 2002). Cyp4d21 demonstrates sex-specific expression patterns in the male head and is recognized as a circadian-regulated gene, aligning with the circadian clock gene network (Fujii and Amrein 2002) that also governs LMD behavior (Kim *et al*. 2012). Notably, it has been proposed that the male-specific transcriptional regulator dsx^M^ and clock genes are required for the circadian regulation of *sxe1* mRNA. Cyp4d21 protein expression is restricted to non-neuronal cells associated with diverse sensory bristles of both the chemo- and mechanosensory systems (Fujii *et al*. 2008). Given that LMD is also mediated by sexually dimorphic circuits and male-specific behaviors, the elucidation of the mechanistic aspects of Cyp4d21 will provide crucial insights into the underlying mechanisms of LMD behavior, thus enhancing our understanding of this complex behavior in *Drosophila*.

## Discussion

In this study, we conducted a comprehensive investigation to delineate the genes that are upregulated in single-housed male *Drosophila* and their associations with mating behaviors (Wang *et al*. 2008). Through the analysis of mutants for these genes, we observed that the majority of them significantly influenced LMD behavior but had minimal effects on CL (Figs. 1 and 2, Supplementary Figs. 1 and 2). This indicates that these genes primarily regulate the mating duration rather than the initiation of mating or copulation behavior. Furthermore, our single-cell RNA sequencing data revealed that the expression of these LMD-related genes predominantly overlaps with that of male-specific genes such as *dsx* and *Cyp6a20* in specific cell populations, especially in cone cells (Supplementary Figs. 1 and 2). The functional validation of a subset of these genes further confirmed their roles in mediating LMD behavior. Expression of these LMD genes, including *Cyp6a20*, *Cyp4d21*, and *CrzR*, is enriched in cone cells, with disruptions in cone cell-specific expression of *CrzR* and *Cyp4d21* leading to disrupted LMD (Figs. 4 and 5). Significantly, the metabolic gene *Cyp6a20*, known to modulate aggressive behavior, has been revealed as a critical regulator of LMD in male *Drosophila*. The expression of *Cyp6a20* within cone cells is found to be indispensable for the proper expression of LMD, highlighting the pivotal role of this gene in both aggression and mating behaviors (Fig. 5 and Supplementary Fig. 5). Moreover, we identified a novel gene, *CG10026/macewindu*, which is required for LMD when overexpressed in cone cells (Fig. 4). Collectively, our findings underscore the critical role of cone cells as a pivotal site for the expression of genes involved in the regulation of LMD behavior. These insights provide a valuable foundation for further exploration into the intricate mechanisms underlying this complex behavior in *Drosophila* (Fig. 5p).

Although the multimodal sensory integration for courtship latency is deeply investigated (Griffith and Ejima 2009a), the sensory modality affecting CL is not highly investigated regardless of its importance on male’s reproductive fitness. A previous study reported that wild-type males exhibit decreased copulation latency toward oenocyte-removed females compared with normal females (Billeter *et al*. 2009), indicating that female pheromones mediate copulation latency of males (Dweck *et al*. 2015). Although it is well known that CL of *white* mutant males is significantly delayed compared to wild-type males, there is no report of how this effect is related to the sensory modality of male flies (Xiao *et al*. 2017). Further research is necessary to fully elucidate the role of CL in the complex dynamics of mating behavior.

Cone cells are specialized epithelial cells found within the ommatidia, the structural units of the compound eye in *Drosophila melanogaster,* and are responsible for the secretion of the lens material that forms the corneal facet. These cells are integral to the fly’s visual system, contributing significantly to photoreceptor performance. Furthermore, evidence suggests that cone cells also perform glia-like support functions within the *Drosophila* eye, facilitating the maintenance and functionality of the photoreceptor cells (Yamaguchi *et al*. 2008). These specialized photoreceptor cells are located in the dorsal region of the fly's compound eye and are responsible for detecting and processing a wide range of wavelengths, thereby enabling the discrimination of different colors (Cook *et al*. 2003; Morante and Desplan 2008; Schnaitmann *et al*. 2013; Behnia and Desplan 2015). Investigating how the differential expression of genes within cone cells modulates visually oriented LMD, aggression, and courtship (Kohatsu and Yamamoto 2015) in male *Drosophila* would be an intriguing scientific pursuit. While the role of the *white* gene mutation in augmenting aggressive behavior has been genetically mapped, the nexus between visual perception and aggression remains unexplored compared to the well-documented pheromonal influences on aggression (Wang and Anderson 2010; Zwarts *et al*. 2012). This report introduces a novel approach by examining the correlation between cone cell morphology and an aggression index across diverse genotypes of flies. Such an analysis could elucidate the genetic and neuronal interplay between visual processing and the expression of aggressive behavior in animals. This inquiry has the potential to unravel the intricate interplay between visual sensory cues, gene expression patterns, and complex behavioral outcomes.

The observation that cone cells predominantly express the *doublesex (dsx)* gene, while the *fruitless (fru)* gene is not significantly expressed, carries important implications for understanding sexual dimorphism in *Drosophila*. Cone cells, which are non-neuronal and provide structural support to the compound eye, appear to primarily express *dsx* for the differentiation of physical sex characteristics rather than fru. This is consistent with the known roles of DSX and FRU in flies: *dsx* has a broader expression pattern and is crucial for coordinating sexual differentiation in both neural and non-neural tissues, while *fru* is mainly expressed in the nervous system and is essential for male-specific behaviors and courtship (Yamamoto *et al*. 1998; Rideout *et al*. 2010; Meier *et al*. 2013; Yamamoto and Koganezawa 2013; Pan and Baker 2014; Zhou *et al*. 2014; Duckhorn *et al*. 2022). The absence of *fru* expression in cone cells suggests that these cells do not play a direct role in the neural processes underlying male courtship or aggressive behaviors. Instead, their expression of *dsx* likely contributes to the development of male-specific physical traits (Yamamoto *et al*. 1998; Siwicki and Kravitz 2009; Rideout *et al*. 2010; Dauwalder 2011). The fact that LMD behavior is governed by genes expressed in cone cells, such as *Cyp6a20* and *Cyp4d21*, highlights the complex interplay between neural and non-neuronal mechanisms in regulating male sexual behaviors. These findings underscore the importance of *dsx*-positive cone cells as a critical site for the expression of genes involved in the regulation of LMD behavior, shedding light on the diverse roles of *dsx* and *fru* in sexual dimorphism in *Drosophila* (Kohatsu and Yamamoto 2015).

The intricate network of cells that constitutes the nervous system is a marvel of biological complexity, with various cell types collaborating to facilitate the transmission and processing of information. Among these, neurons are traditionally viewed as the primary effectors of neural function. However, recent advancements in neurobiology have underscored the pivotal role of non-neuronal cells, such as glial cells, in supporting and regulating neuronal activity. In the fruit fly *Drosophila melanogaster*, cone cells, a specialized type of photoreceptor cell within the compound eye, exhibit a function analogous to that of glial cells in the central nervous system (CNS). Cone cells provide essential support to the neural circuitry underlying visual processing and behavior, and they do so through a sophisticated interplay of metabolic regulation and gene expression (Kumar *et al*. 2015; Stahl *et al*. 2017; Muraoka *et al*. 2018). The metabolic demands of neural tissue are substantial, and cone cells are integral to meeting these demands in the context of the visual system. They express a repertoire of metabolic genes that are pivotal for the synthesis and breakdown of key metabolites, such as neurotransmitters and energy-rich molecules. This metabolic gene expression in cone cells is finely tuned to match the fluctuating energy requirements of the visual circuitry, ensuring that neurons can maintain their activity levels and respond appropriately to environmental stimuli. The findings from this research may serve as a foundation for future studies on the role of non-neuronal cells in sensory processing and may have implications for our understanding of glial–neuronal interactions in health and disease across various species.

## Supplementary Material

jkae255_Supplementary_Data

## Data Availability

Strains are available upon request. The authors affirm that all data necessary for confirming the conclusions of the article are present within the article, figures, and tables. Supplemental material available at G3 online.
