# Supplementary material for: Genetic screening reveals cone cell-specific factors as common genetic targets modulating rival-induced prolonged mating in male Drosophila melanogaster
Source: G3 (Bethesda). 2024 Nov 4;15(1):jkae255. doi: 10.1093/g3journal/jkae255 (PMC11708226; doi:10.1093/g3journal/jkae255)
Supplement: jkae255_Supplementary_Data [file jkae255_supplementary_data.zip › Supplemental_Figure_Legends_G3-2024-405485.docx]

**Supplemental Figure Legends**

**Figure S1.** Single-housing upregulated genes related to LMD co-express with *dsx* and *Cyp6a20* in specific head cell populations.

a-b) Schematic diagram of behavioral assay. Naïve (group-housed) and singly reared males were exposed to females for 1 hour in a mating chamber. Only successful matings were analyzed. Copulation initiation, completion, and duration were recorded (a), Copulation latency (CL) was quantified by recording the time from the initiation of mating and mating duration (MD) is the time from contact to completion of a mating (b).

c-f) MD and CL of *fbl^1^/+* (c and d) and *Pri^1^/+* (e and f) males in LMD conditions. ns represents non-significant difference (unpaired *t*-test).

g–t) tSNE plots within the head colored by gene expression: *fbl* (g), *Pri* (h), *spin* (i), *b6* (j), *bgm* (k), *wdp* (l), *Pisd* (m), *Jhe* (n), *Nplp1* (o), *Rya-R* (p), *CAP* (q), *pkg21D* (r), *Hexo1* (s) and *fru* (t) in red, *dsx* in green, and *Cyp6a20* in blue. Each tSNE visualization depicts the coexpression patterns of genes, with each color corresponding to the genes listed on the left, right, and bottom of the plot. The tissue name, as referenced on the Fly SCope website is indicated in the upper left corner of the tSNE plot. Dashed lines denote the significant overlap of cell populations annotated by the respective genes.

**Figure S2.** Male-male interaction-regulated genes co-express with *Cyp6a20* in cone cells.

a–d) MD and CL of *CG8654^MB09631^/+* (a and b) and *CG11200^d02302^*/+ (c and d) males in LMD condition. **, *P* < 0.01 (unpaired *t*-test).

e–l) tSNE plots within the head colored by gene expression: *CG8654* (e), *CG11200* (f), *CG1544* (g), *CG10026* (h), *CG12560* (i), *CG31689* (j), *CG31075* (k), and *CG33120* (l) in red, *dsx* in green, and *Cyp6a20* in blue.

m-q) tSNE plots within neuron (m), the glial cell (n), the sensory organ cells (k), epithelial cells (o), and excretatory system (p) colored by cell types or gene expression: cone cells in red, *dsx* in green, and *Cyp4d20* in blue.

**Figure S3.** Cone cell genes *GMR* and *prospero* co-express with *Cyp6a20* and *dsx*.

a-b) *GMR-GAL4* drives expression of GFP reporter (*GMR*>GFP) in the adult male and female eyes.

c-d) *CrzR* is expressed in *gl* (*GMR*) and *prospero* that overlaps *Cyp6a20* within head. tSNE plots colored by cell types or gene expression: *CrzR* in red, *Cyp6a20* in green, *gl* (*GMR*) (c) and *prospero* (d) in blue.

e) *cone cell* is expressed in *prospero* that overlaps *dsx* within epithelial cell. tSNE plots colored by cell types or gene expression: *cone cells* in red, *dsx* in green, *prospero* in blue.

f) *pros-GaL4* drives expression of GFP reporter (*pros*>RFP) in the adult male eyes. Cone cells of male flies expressing *GAL4^pros^* together with *UAS-mCD8RFP* were immunostained with anti-cut (green) antibody.

g) *pros-GaL4* drives expression of GFP reporter (*pros*>GFP) in the adult male eyes. Cone cells of male flies expressing *GAL4^pros^* together with *UAS-mCD8GFP* were immunostained with anti-cut (red) antibody and DAPI (blue). Scale bars represent 100 μm. Dashed circles indicate the region of interest.

h-i) MD assay of *GMR-GAL4/+* males (h) and spin-RNAi/+ males (i) in LMD condition. **, *P* < 0.01 (unpaired *t*-test). **, *P* < 0.01 (unpaired *t*-test); ****, *P* < 0.0001 (unpaired *t*-test).

**Figure S4.** *CG10026*, *Crys* and *Retinin* in cone cells are crucial for inducing LMD behavior.

a) MD assay of *CG10026^d02517^/+* males in LMD condition. **, *P* < 0.01 (unpaired *t*-test).

b) tSNE plots within the epithelial cells colored by cell types or gene expression:*CG10026* in red, cone cells in green, and *dsx* in blue.

c-d) tSNE plots within head colored by cell types or gene expression:*CG10026* in red, cone cells in blue, *elav* (c) and *repo* (d) in green.

e-g) Features of *CS*, *W^1118^* and *Crys^MI07191^* in adult male eyes. Cone cells of male flies *CS*, *W^1118^* and *Crys^MI07191^* were immunostained with anti-cut (red) and anti-elav (green) antibodies and DAPI (blue). Scale bars represent 100 μm.

h-i) MD and CL assays of *Crys^MI07191^/ Crys^MI07191^* males in LMD condition. **, *P* < 0.01 (unpaired *t*-test).

j-k) LMD and CL assays for GAL4-mediated knockdown of retinin *via retinin-RNAi* using the *GMR-GAL4* driver. ns represents non-significant difference (unpaired *t*-test).

l-m) MD assays of *pros-GAL4/+* males (l) and *CrzR-RNAi/+* (m) males in LMD condition. **, *P* < 0.01 (unpaired *t*-test); ****, *P* < 0.0001 (unpaired *t*-test).

**Figure S5.** Male-male interaction-regulated genes *Cyp6a20*, *ebony* and *Cyp4d21* have differential effect on mating duration.

a) LMD and SMD assays of *Cyp6a20^e02611^/+* males. ***, *P* < 0.001; ****, *P* < 0.0001 (unpaired *t*-test).

b) CL assay of *ebony^1^/ebony^1^* in LMD and SMD conditions. **, *P* < 0.01 (unpaired *t*-test). c-d) MD and CL assay of *Cyp4d21^MB11663^/+* males in LMD condition. ****, *P* < 0.0001 (unpaired *t*-test).

e-f) LMD assays of *Cyp6a20-RNAi/+* males (e) and *Orco-GAL4/+* males (f). ***, *P* < 0.001; ****, *P* < 0.0001 (unpaired *t*-test).

g) SMD behavior assay diagram. On day 4 of eclosion, 2:1 ratios of DF females and male flies were mated. On day 5, males were mated with Df again, anesthetized, and recovered at 25°C for 1 hour. Mating was initiated by removing the clear film and observed for 1 hour. Only successful pairs were analyzed, with mating start and end times recorded to the nearest 10 seconds.
