# Supplementary figures and images for: Genetic screening reveals cone cell-specific factors as common genetic targets modulating rival-induced prolonged mating in male Drosophila melanogaster
Source: G3 (Bethesda). 2024 Nov 4;15(1):jkae255. doi: 10.1093/g3journal/jkae255 (PMC11708226; doi:10.1093/g3journal/jkae255)

**a** LMD behavior assay

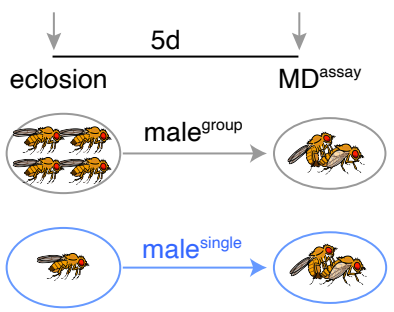

**b** Courtship latency (C.L.) vs. Mating duration (M.D.)

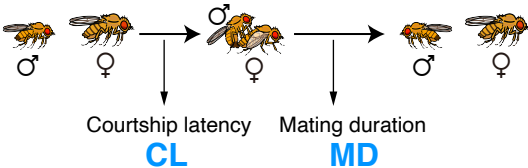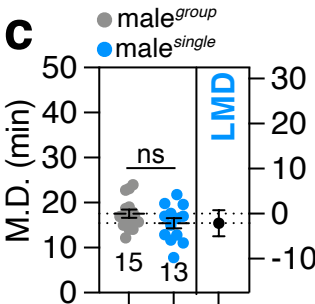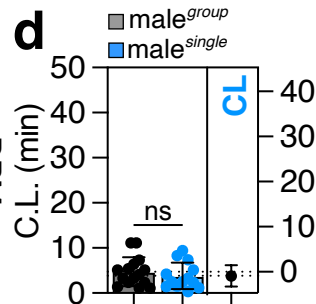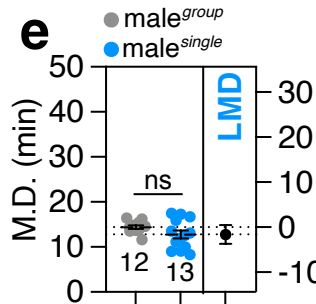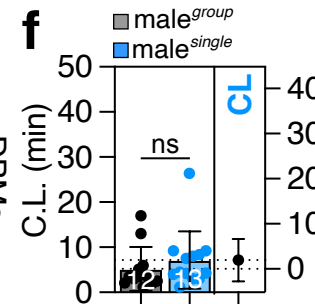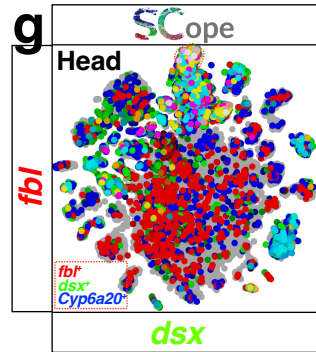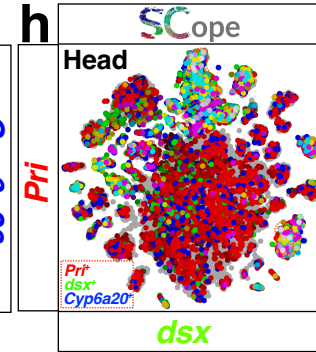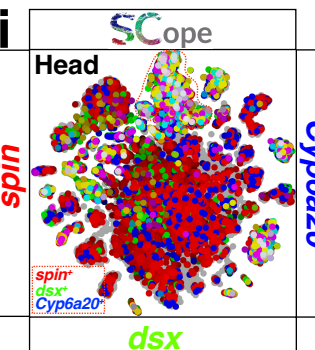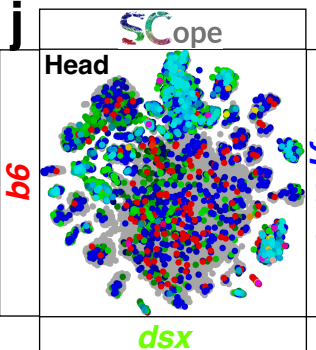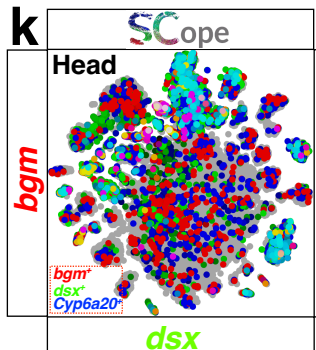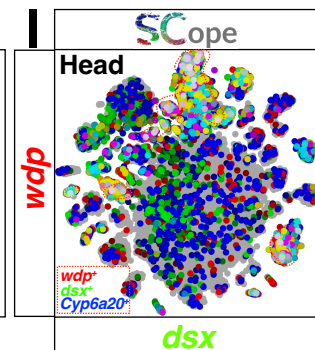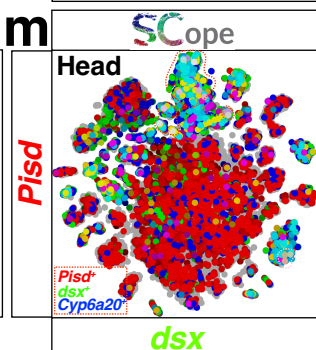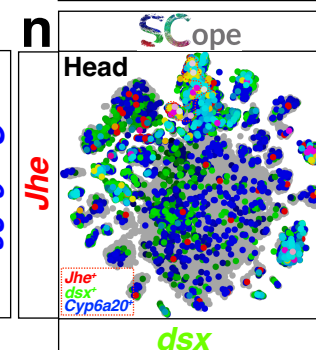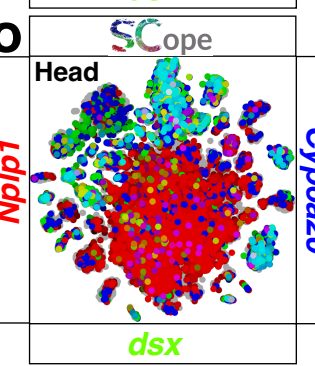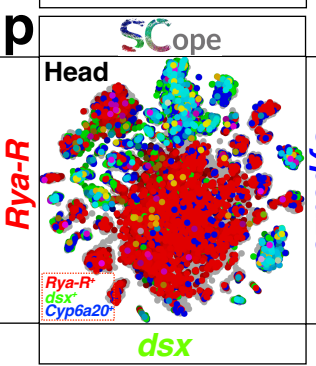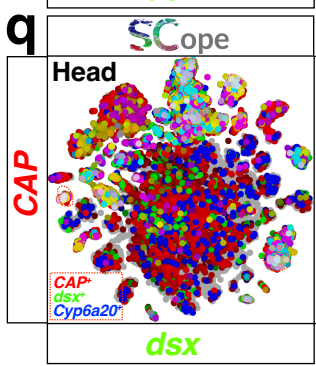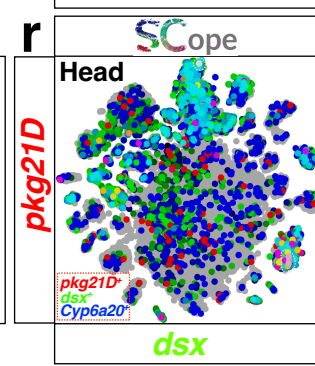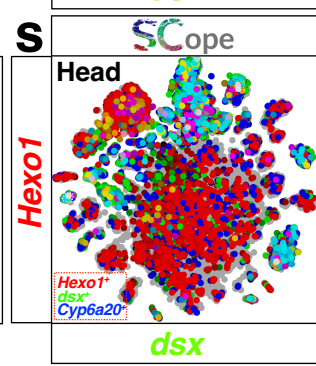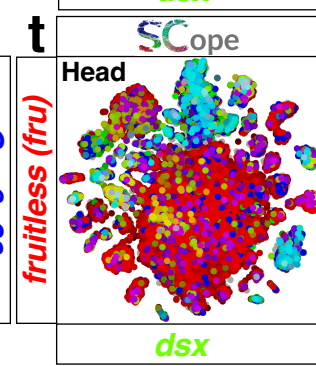

Supplement: jkae255_Supplementary_Data [file jkae255_supplementary_data.zip › Figure_S1_G3-2024-405485.pdf]

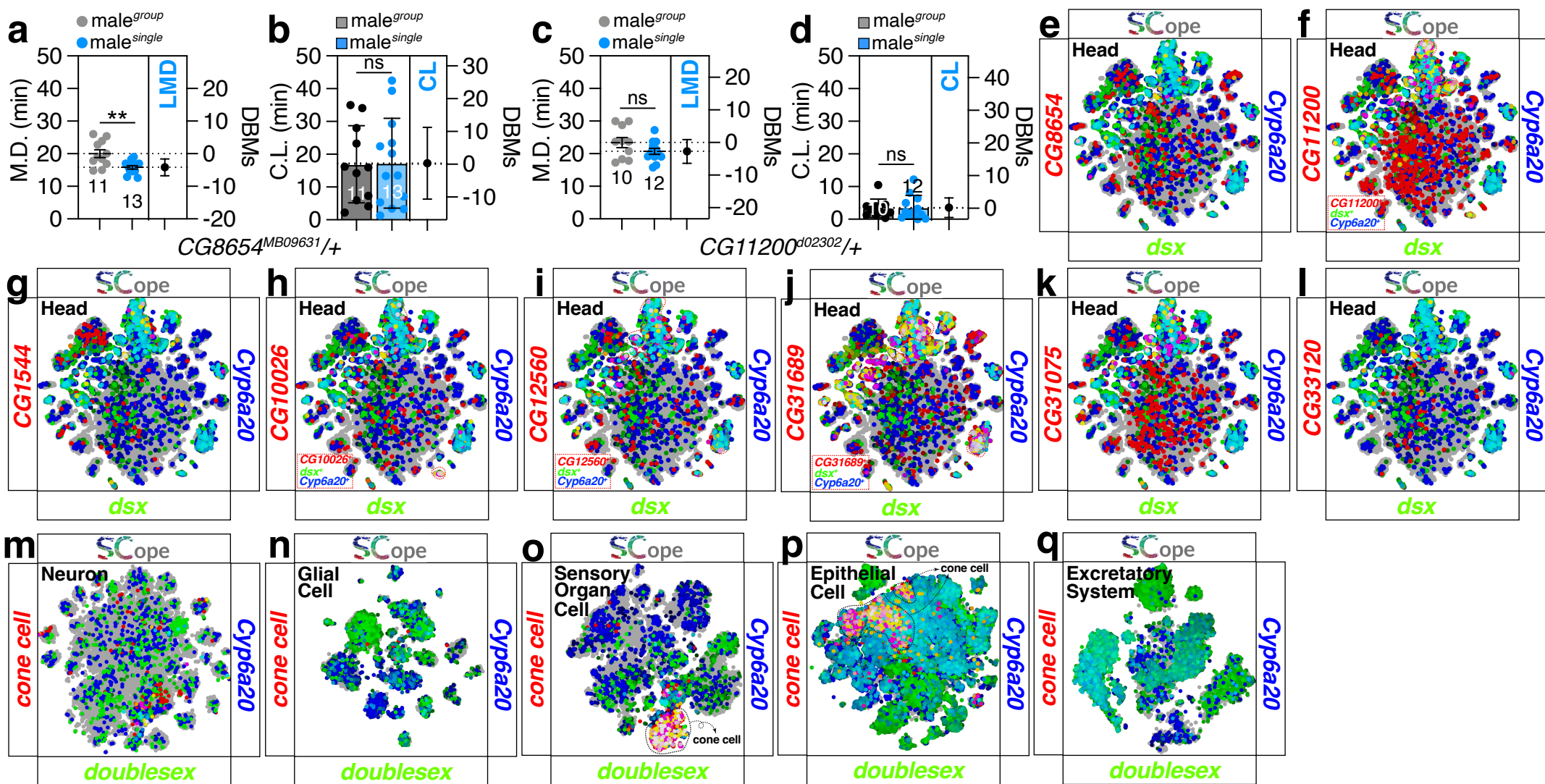

Supplement: jkae255_Supplementary_Data [file jkae255_supplementary_data.zip › Figure_S2_G3-2024-405485.pdf]

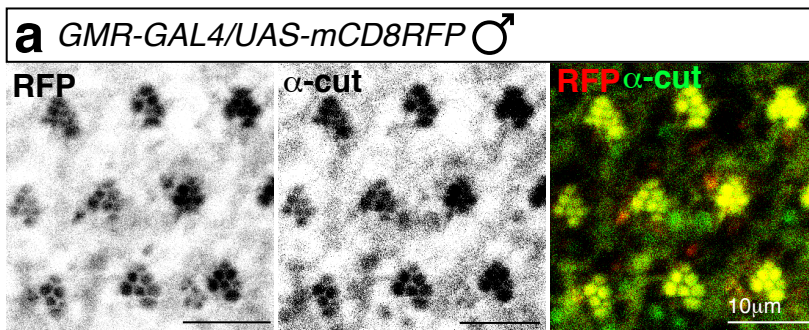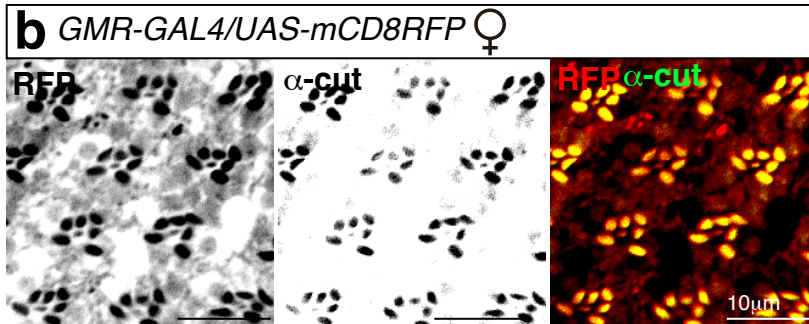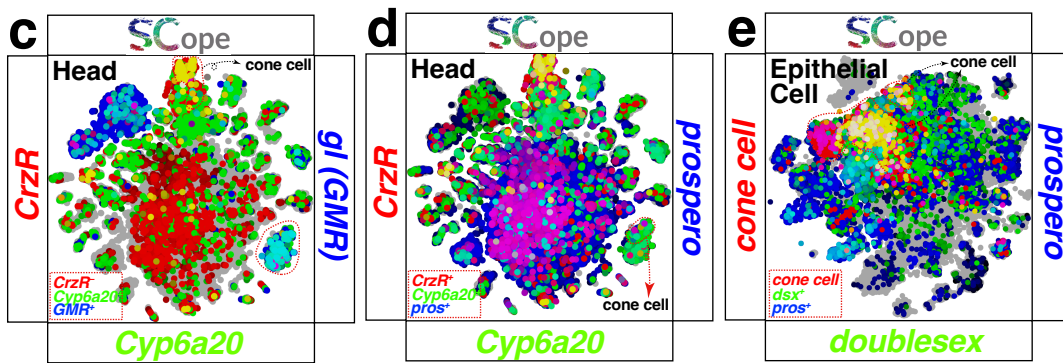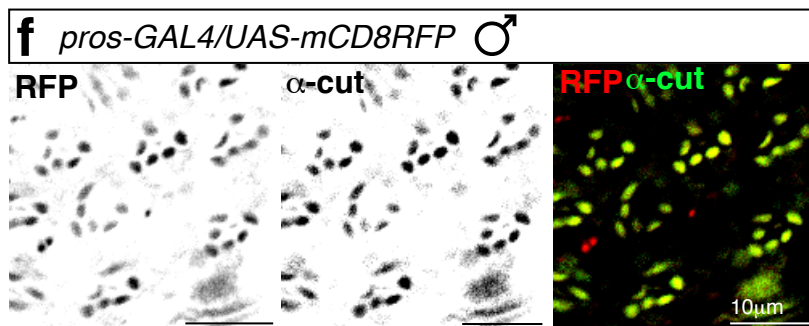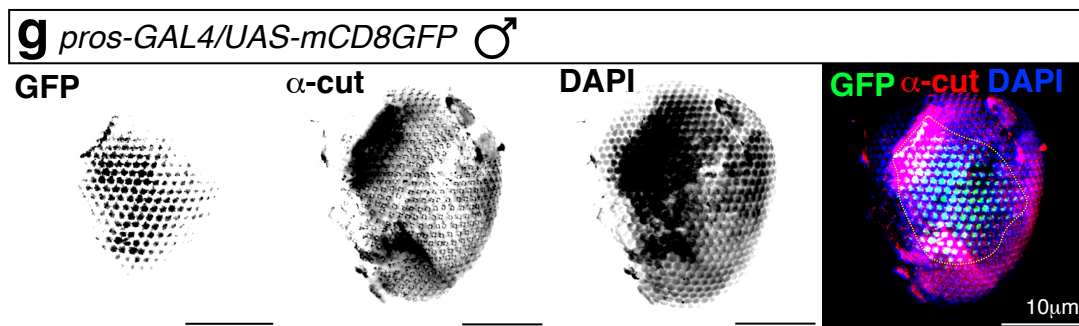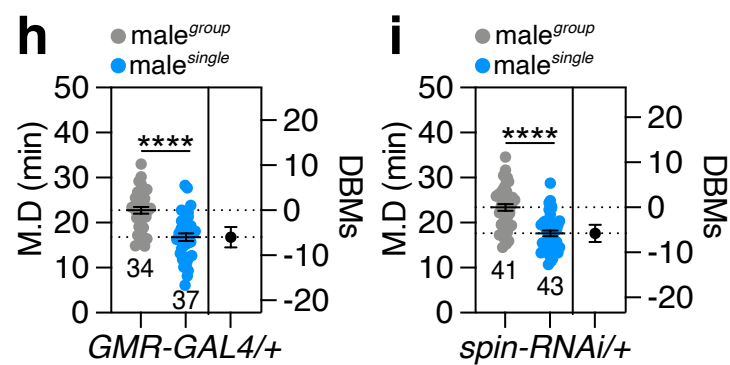

Supplement: jkae255_Supplementary_Data [file jkae255_supplementary_data.zip › Figure_S3_G3-2024-405485.pdf]

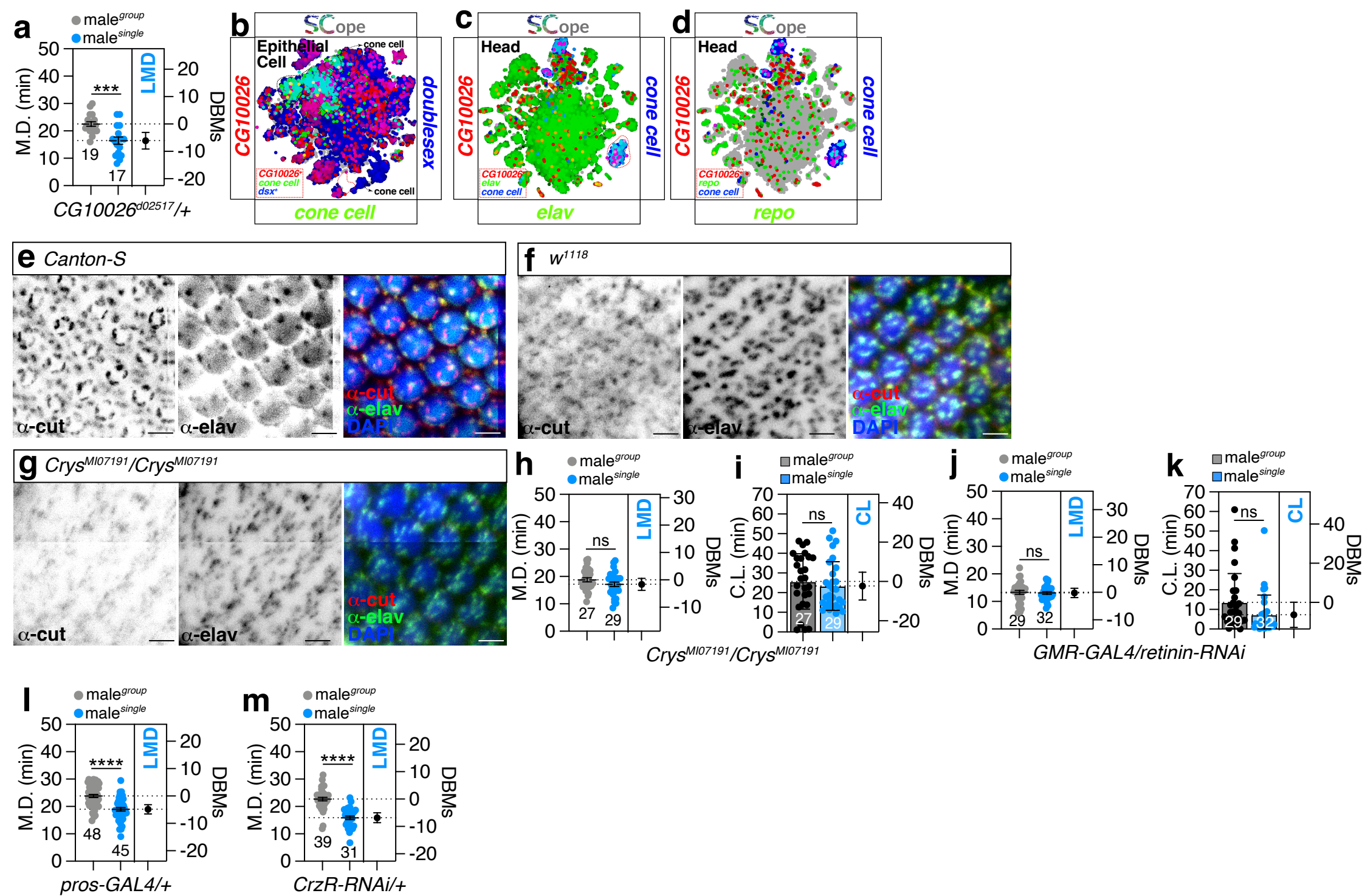

Supplement: jkae255_Supplementary_Data [file jkae255_supplementary_data.zip › Figure_S4_G3-2024-405485.pdf]

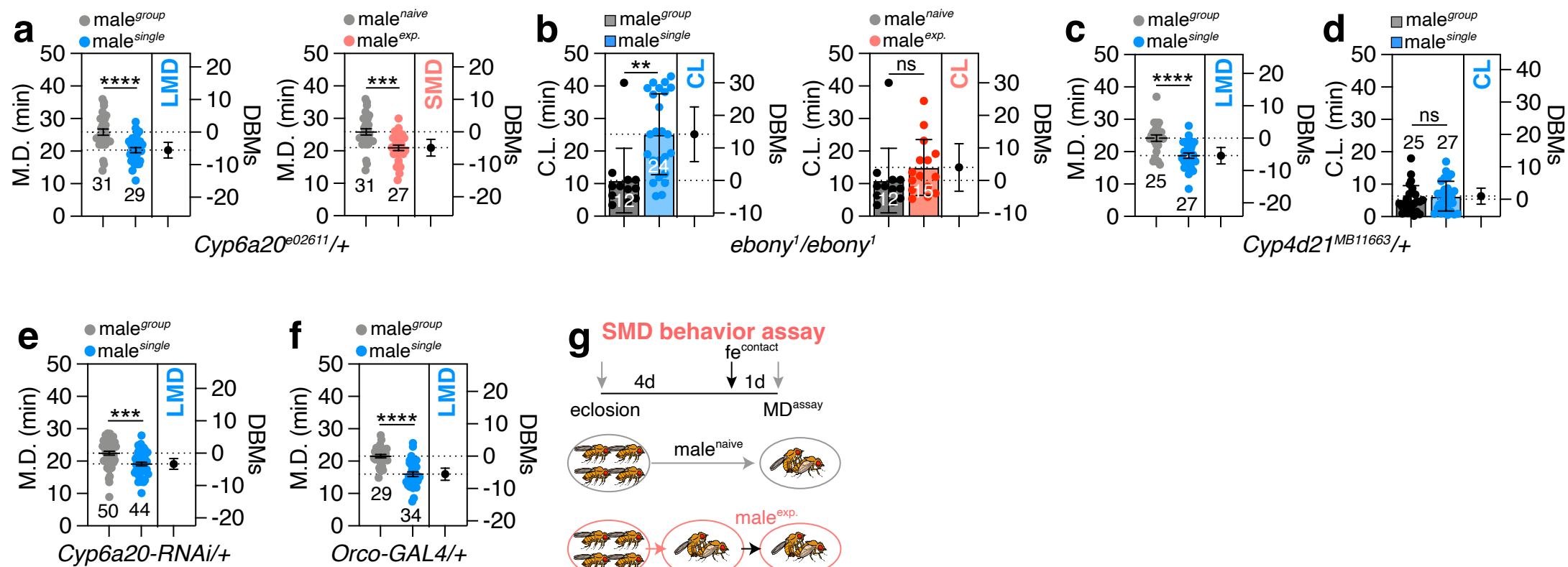

Supplement: jkae255_Supplementary_Data [file jkae255_supplementary_data.zip › Figure_S5_G3-2024-405485.pdf]
